# The Diseases of the Pancreas, and Their Homœopathic Treatment

**Published:** 1882-08

**Authors:** 


THE DISEASES OF THE PANCREAS, AND THEIR HOMŒOPATHIC TREATMENT. By Drs. Farrington, Karndœrfer, Morgan and Thomas. Chicago: Duncan Bros. 1882.

This essay was prepared originally by the above-mentioned members of the Philadelphia County Homœopathic Society, as a contribution from that Society to the Pennsylvania State Society, from whose transactions it is now reissued. It gives a thorough history of the pancreas, its anatomy, physiology, etc.; the therapeutic portion is very scant. But, fortunately for us, we base our prescriptions upon the totality of symptoms, general and local, hence a paucity of therapeutics as relating to any one organ does not greatly lessen our efficiency to heal. The pancreas cannot be "sick" without its possessor being so also.

